# Docosatrienoic Acid Inhibits Melanogenesis Partly through Suppressing the Intracellular MITF/Tyrosinase Axis

**DOI:** 10.3390/ph17091198

**Published:** 2024-09-11

**Authors:** Kyoung Mi Moon, Min-Kyeoun Lee, Su-Yeon Park, Jaeseong Seo, Ah-reum Kim, Bonggi Lee

**Affiliations:** 1 Department of Food Science and Nutrition, Pukyong National University, Busan 48513, Republic of Korea; omkksm@pknu.ac.kr (K.M.M.); lmk1905@korea.kr (M.-K.L.); suzan@pukyong.ac.kr (S.-Y.P.); syj5528@pukyong.ac.kr (J.S.); kimar0107@pukyong.ac.kr (A.-r.K.); 2 Biotechnology Research Division, National Institute of Fisheries Science, Busan 48513, Republic of Korea; 3 Department of Smart Green Technology Engineering, Pukyong National University, Busan 48513, Republic of Korea; 4 Marine Integrated Biomedical Technology Center, The National Key Research Institutes, Pukyong National University, Busan 48513, Republic of Korea

**Keywords:** melanogenesis, docosatrienoic acid, fatty acid, skin aging

## Abstract

Melanogenesis, essential for skin photoprotection and pigmentation, can lead to disorders like melasma and hyperpigmentation, which are challenging to treat and affect quality of life. Docosatrienoic acid (DTA), a polyunsaturated omega-3 fatty acid, has been identified as a potential regulator of skin aging. This study investigates DTA’s effects on melanogenesis and its underlying molecular mechanisms using in silico and in vitro analyses. SwissSimilarity analysis revealed that DTA shares close structural similarities with known anti-melanogenic lipids, suggesting it may inhibit melanogenesis in similar manners. Our results demonstrated that DTA reduces melanin content and intracellular tyrosinase activity in B16F10 cells, significantly downregulating the mRNA expression of *tyrosinase*, *TRP-1*, and *TRP-2* by inhibiting MITF translocation to the nucleus. While DTA exhibited mild inhibitory effects on mushroom tyrosinase activity and antioxidant properties at higher concentrations, direct inhibition of tyrosinase is likely not the primary mechanism, as the observed anti-melanogenic effects occurred at much lower concentrations compared to those required for direct tyrosinase inhibition. Together, DTA-mediated modulation of MITF and tyrosinase mRNA expression offers a novel approach to treating hyperpigmentation. DTA’s potential extends into the cosmetic industry, enhancing product stability, functionality, and aesthetics. Further research is needed to explore DTA’s broader applications in skincare and cosmetic formulations.

## 1. Introduction

Melanogenesis, while essential for protecting the skin from ultraviolet (UV) radiation and contributing to skin and hair pigmentation, can have several harmful effects under certain conditions. Disorders like melasma and post-inflammatory hyperpigmentation result in dark, discolored patches on the skin, which are often challenging to treat and affect individuals’ quality of life. Excessive UV exposure linked to melanogenesis can lead to skin cancers, including melanoma, by causing DNA damage in melanocytes. Additionally, chronic UV exposure also results in age-related pigmentation such as lentigines and contributes to photoaging, characterized by uneven skin tone and an aged appearance [1,2]. Furthermore, visible pigmentation disorders can significantly impact self-esteem and mental health, leading to emotional distress [3].

In addition to these risks, genetic disorders related to melanin production, such as albinism, which involves a lack of melanin, make individuals highly susceptible to sunburn and skin cancers, while vitiligo leads to depigmented patches on the skin, causing psychological and social effects. These issues underscore that, although melanogenesis is crucial for photoprotection, its dysregulation can lead to various skin disorders and increased cancer risk, highlighting the need for effective treatments and preventive measures [1,2,4].

Recent research has further elucidated the complex roles that melanin plays beyond mere photoprotection. For example, melanin is now understood to be involved in the modulation of immune responses, neuroprotection, and the regulation of reactive oxygen species (ROS) [5]. These findings underscore the importance of maintaining balanced melanin production, as both overproduction and underproduction can lead to significant physiological consequences.

Melanogenesis is a complex process that occurs within specialized organelles called melanosomes in melanocytes and involves multiple enzymatic reactions. The key enzyme, tyrosinase, catalyzes the conversion of tyrosine to dopaquinone, initiating melanin synthesis [1,6]. This process is tightly regulated by various factors, including tyrosinase-related proteins (TYRP1 and TYRP2), the microphthalmia-associated transcription factor (MITF), and multiple signaling pathways. MITF is particularly significant as it regulates the expression of essential melanogenic enzymes and proteins, thus controlling melanin production [7]. Understanding and regulating these molecular mechanisms is essential for developing therapeutic interventions for pigmentation disorders such as vitiligo, melasma, and hyperpigmentation.

Ultraviolet (UV) radiation, particularly UVB, is a primary environmental factor that induces melanin production. However, UV radiation’s effects extend beyond melanin production; it also interacts with the neuroendocrine and immune systems, influencing overall human physiology. This intricate interplay, known as “photo-neuro-immuno-endocrinology”, reflects the complex regulatory mechanisms by which UV radiation affects the body, brain, and immune system [8]. These interactions highlight the importance of understanding UV radiation’s broader impacts when considering the regulation of melanogenesis.

Melanogenesis is a complex process influenced by various factors, including recent research highlighting the significant role of fatty acids in modulating this process. Fatty acids are known for their functions in energy metabolism and cellular signaling, and they are also recognized for their impact on melanin production [9,10]. These lipids, classified by their chain length and degree of unsaturation, exhibit distinct effects on melanogenesis. Saturated fatty acids, such as palmitic acid, have been shown to stimulate melanin production, whereas polyunsaturated fatty acids, like linoleic acid, have inhibitory effects on this process [11]. The interaction between fatty acids and melanogenesis has important implications in fields such as dermatology, cosmetology, and oncology [12,13,14]. Insights gained from studying these interactions may lead to novel therapeutic strategies for addressing pigmentation disorders and melanoma.

Docosatrienoic acid (DTA), also known as 22:3(n-3), is a polyunsaturated omega-3 fatty acid with a long carbon chain comprising 22 carbon atoms and three cis double bonds. These double bonds are typically located at the 7th, 10th, and 13th carbon positions from the omega end, contributing to the molecule’s flexibility and fluidity in biological membranes. This structural configuration may allow DTA to play a crucial role in maintaining cell membrane integrity and enhancing membrane fluidity [15,16]. However, despite its potential significance, the biological functions of this lipid have not been thoroughly investigated. In this study, we examined whether this fatty acid regulates melanogenesis and explored its underlying molecular mechanisms. To achieve this, we conducted both in silico and in vitro analyses to investigate the anti-melanogenic properties of DTA.

## 2. Results

### 2.1. Structural and Functional Analysis of Docosatrienoic Acid (DTA)

SwissSimilarity is a powerful web-based tool designed to facilitate the rapid screening of extensive libraries, including drugs, bioactive small molecules, commercially available compounds, and an ultra-large collection of virtual compounds that can be readily synthesized from commercially available reagents. It enables efficient identification of potential bioactive compounds using molecular fingerprints and both superpositional and fast non-superpositional 3D shape similarity approaches. These methods allow to compare and identify similar compounds quickly, aiding in drug discovery, molecular mechanism studies, and lead compound optimization [17].

**Figure 1:**
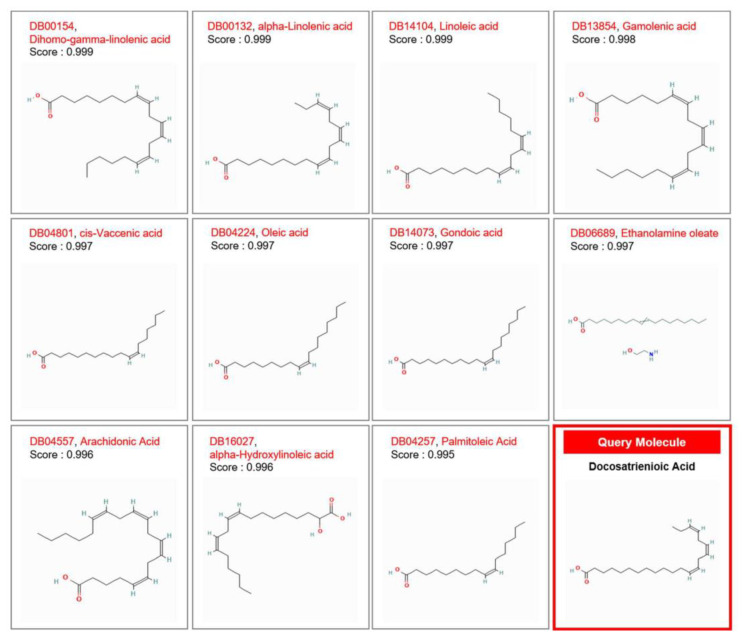
Structural analysis of docosatrienoic acid (DTA) using SwissSimilarity. SwissSimilarity was used to compare docosatrienoic acid (DTA) with structurally similar lipids to predict its potential functions in the skin. SwissSimilarity enables the rapid analysis of extensive libraries, including drugs, bioactive small molecules, commercially available compounds, and an immense collection of virtual compounds that can be easily synthesized from commercially accessible reagents. The virtual screening process utilizes molecular fingerprints, as well as both super positional and non-super positional 3D shape similarity methods. The chemical structure images for the similar lipids were obtained from PubChem.

Although previous research indicated that DTA accumulates in human fibroblast phospholipids when cultured in a basal nutrient medium (MCDB 110) with reduced fetal bovine serum levels (0.4%) [18], the role of this lipid in skin aging is not well understood. To explore its potential functions in the skin, we used SwissSimilarity to compare DTA with structurally similar lipids. The analysis revealed that DTA shares a high similarity with several lipids: dihomo-gamma-linolenic acid (similarity score 0.999), alpha-linolenic acid (0.999), linoleic acid (0.999), gamma-linolenic acid (0.998), cis-vaccenic acid (0.997), oleic acid (0.997), gondoic acid (0.997), ethanolamine oleate (0.997), arachidonic acid (0.996), alpha-hydroxylinoleic acid (0.996), and palmitoleic acid (0.995) (Figure 1). Of these lipids, palmitoleic acid, alpha-linolenic acid, and linoleic acid [10] exhibited anti-melanogenic properties (Figure 1) [19]. Although found in minimal amounts in mammalian tissues, dihomo-gamma-linolenic acid and its metabolites are increasingly recognized as crucial mediators of inflammation, potentially playing important roles in specific disease conditions including obesity, type 2 diabetes, cardiovascular diseases, hepatic diseases, cancers, gastrointestinal diseases, arthritis, bronchial asthma, and atopic dermatitis [20]. Given the structural similarity of DTA to these lipids, we hypothesized that it might also inhibit melanogenesis. To test this hypothesis, we conducted a series of in vitro and cell-free experiments to evaluate the potential anti-melanogenic effects of DTA.

### 2.2. Effects of DTA on Melanogenesis and Tyrosinase Activity in B16F10 Cells

**Figure 2:**
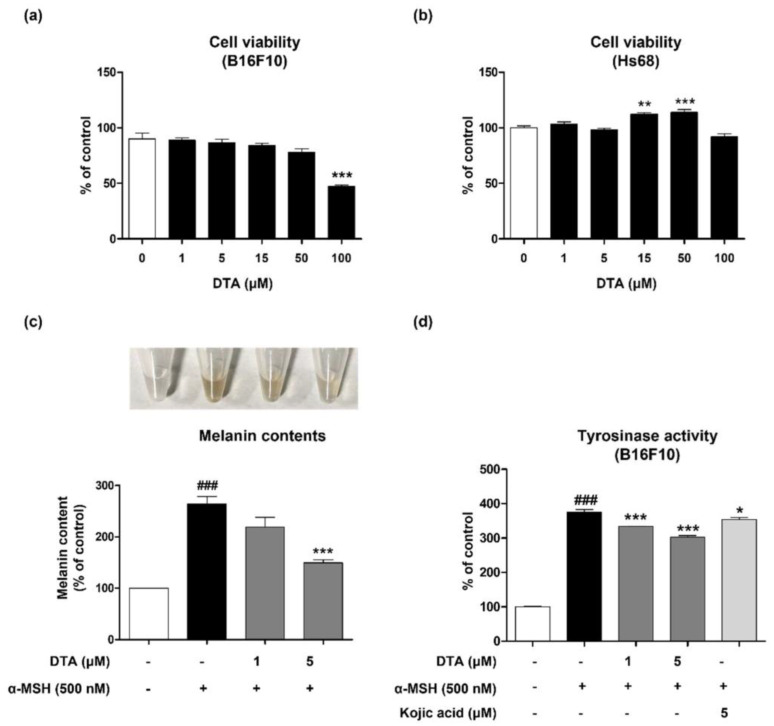
Effects of DTA on melanogenesis in α-MSH-induced B16F10 melanoma cells. Cell cytotoxicity was assessed in (**a**) B16F10 and (**b**) HS68 cells treated with various concentrations of DTA (1–100 μM) for 24 h (n = 4/group). (**c**) Intracellular melanin content and (**d**) tyrosinase activity were measured in B16F10 cells pretreated with various concentrations of DTA (1, 5 μM) and kojic acid (5 μM) for 1 h, followed by stimulation with α-MSH (500 nM) for 6 days (n = 4/group). Results are presented as mean ± SEM. ### *p* < 0.001 compared with the non-treated control group and * *p* < 0.05, ** *p* < 0.01, *** *p* < 0.001 compared with the α-MSH-treated group.

To investigate the effects of DTA on melanogenesis, we first evaluated cytotoxicity in various skin cell lines, including B16F10 cells and HS68 cells. DTA showed no toxicity in B16F10 cells up to 15 µM, whereas toxicity was observed at 100 µM in HS68 cells (Figure 2a,b). Based on these findings, we proceeded to assess melanin content in B16F10 cells. Initially, cells were pre-treated with 1 µM and 5 µM DTA, followed by induction of melanin synthesis using α-MSH for 6 days, starting one hour post-pre-treatment. Results indicated that melanin content increased by 250% with 500 nM α-MSH compared to the control, followed by a concentration-dependent decrease (Figure 2c). Notably, treatment with 5 µM DTA reduced melanin content by up to 150% compared to that of the α-MSH treated group (Figure 2c).

Furthermore, to explore the anti-melanogenic effects and mechanism of DTA, intracellular tyrosinase activity was examined. Tyrosinase activity, which had increased approximately 400% with α-MSH treatment, decreased in a dose-dependent manner with DTA treatment, showing a more pronounced reduction compared to the known anti-melanogenic positive control, kojic acid (Figure 2d). Like the results observed for melanin content, treatment with 5 µM DTA significantly reduced intracellular tyrosinase activity (Figure 2d).

### 2.3. Analysis of the Inhibitory Effects of DTA on Melanogenesis-Related Gene Expression

**Figure 3:**
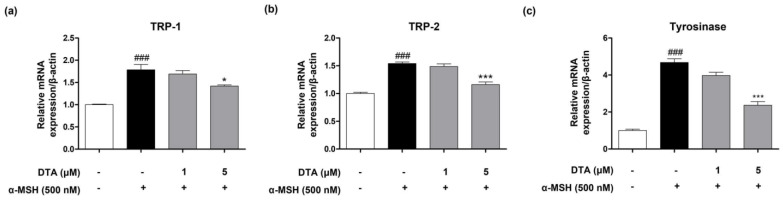
Effects of DTA on the expression of melanogenesis−related genes in B16F10 cells. Real-time PCR analysis of mRNA levels of (**a**) *TRP-1*, (**b**) *TRP-2*, and (**c**) *tyrosinase* in B16F10 cells treated with α-MSH and various concentrations of DTA (1 and 5 μM). The results show a significant downregulation of these genes in a dose-dependent manner with DTA treatment. Results are presented as mean ± standard error of the mean (SEM). ### *p* < 0.001 compared with the non-treated control group, and * *p* < 0.05 and *** *p* < 0.001 compared with the α-MSH-treated group.

To evaluate the impact of DTA on the expression of genes involved in melanogenesis, we measured the mRNA levels of key enzymes such as *TRP-1*, *TRP-2*, and tyrosinase using real-time PCR analysis. Treatment of B16F10 cells with α-MSH significantly increased the mRNA expression levels of *TRP-1*, *TRP-2,* and *tyrosinase* (Figure 3). However, compared to the control group treated only with α-MSH, the DTA-treated group showed a dose-dependent and significant downregulation of these genes (Figure 3).

**Figure 4:**
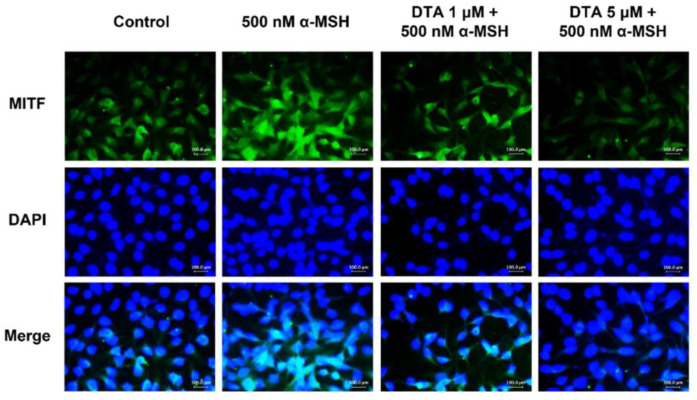
Effects of DTA on MITF nuclear translocation. To evaluate the inhibitory effect of DTA on the nuclear translocation of MITF, immunofluorescence analysis was conducted. B16F10 cells were treated with DTA and observed using fluorescent microscopy. MITF was detected using an anti-MITF monoclonal antibody, followed by an FSD™-conjugated secondary antibody. This analysis shows the localization of MITF and illustrates the impact of DTA on MITF nuclear translocation.

MITF is a transcription factor for *TRP-1*, *TRP-2*, and tyrosinase, playing a crucial role in melanin production. Therefore, we used immunofluorescence staining to determine whether DTA treatment inhibits the translocation of MITF to the nucleus in B16F10 cells. The results showed that treatment with α-MSH alone showed abundant MITF in the nucleus (Figure 4). In contrast, in the DTA-treated group, the translocation of MITF was inhibited in a dose-dependent manner compared to the group treated only with α-MSH (Figure 4). These results suggest that DTA effectively inhibits the expression of genes essential for melanin synthesis, at least partially, by preventing the translocation of the key transcription factor MITF to the nucleus, highlighting its potential as a melanogenesis regulator.

### 2.4. Antioxidant Activity and Tyrosinase Binding Affinity of DTA

**Figure 5:**
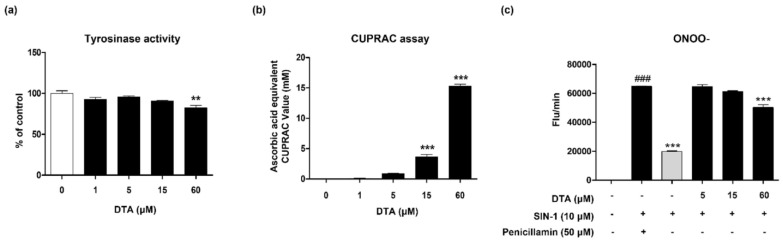
Tyrosinase and antioxidant activities of DTA in a cell−free system. (**a**) Tyrosinase inhibition effect of DTA measured using mushroom tyrosinase. (**b**) Copper ion reduction capacity of DTA determined by CUPRAC analysis, compared to ascorbic acid as a reference. (**c**) Peroxynitrite analysis using DHR123. Data are presented as mean ± standard error of the mean (SEM) (*n* = 3). Results were analyzed using one-way ANOVA followed by Dunnett’s test. ** p < 0.01 and *** p < 0.001 are compared with the control group in (**a**) and (**b**), *### p* < 0.001 is compared with the non-treated control group in (**c**), and *** p < 0.001 is compared with the α-MSH-treated group in (**c**).

Tyrosinase is one of the most important enzymes involved in melanin production. Based on the observed anti-melanogenic effects of DTA in the previous figures, we evaluated whether DTA directly affects tyrosinase activity. Using a cell-free system with mushroom tyrosinase, we treated the enzyme with various concentrations of DTA (5–60 μM) and observed a slight inhibition of tyrosinase activity only at a concentration of 60 μM (Figure 5a).

Oxidative stress can induce melanin production, and antioxidants can inhibit melanin synthesis by reducing oxidative stress. Therefore, in this study, we investigated the antioxidant effects of DTA using the CUPRAC assay and peroxynitrite (ONOO^−^) assay. The results showed that DTA increased the reducing power in the CUPRAC assay at concentrations of 15 μM and 60 μM (Figure 5b). Additionally, SIN-1-induced ONOO- levels were slightly reduced at 60 μM DTA (Figure 5c). Specifically, at higher concentrations (60 μM), DTA demonstrated both an inhibitory effect on mushroom tyrosinase activity and antioxidant activity.

### 2.5. Protein Docking Simulation of DTA with Tyrosinase

**Figure 6:**
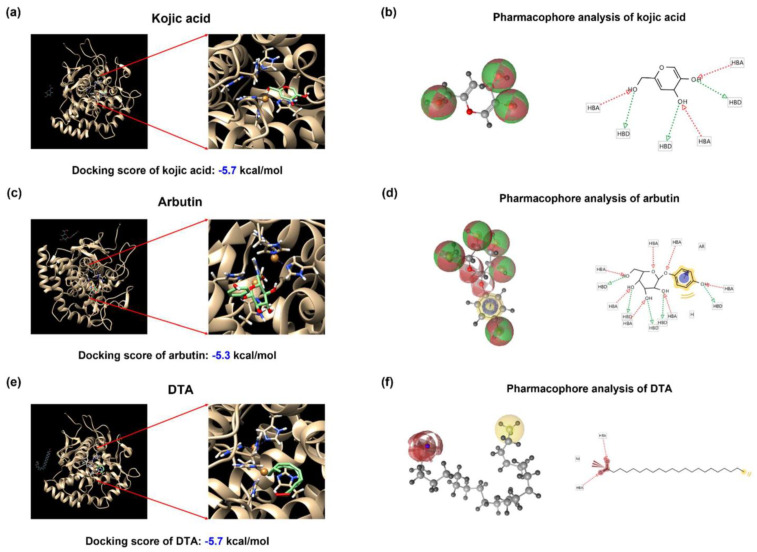
Computational docking analysis of tyrosinase with kojic acid, arbutin, and DTA. Binding affinity analysis between tyrosinase (PDB ID: 2Y9X) and various compounds computational structure prediction for docking simulation between mushroom tyrosinase and various compounds, including (**a**) kojic acid, (**c**) arbutin, and (**e**) dodecanoic acid (DTA). The ligand binding abilities were predicted using AutoDock Vina 1.1.2 software. The indicated boxes show the enlarged images of the binding sites within the tyrosinase structure. The docking scores were: kojic acid (−5.7 kcal/mol), arbutin (−5.3 kcal/mol), and DTA (−5.7 kcal/mol). Pharmacophore analysis was performed to analyze the binding residues of (**b**) kojic acid, (**d**) arbutin, and (**f**) DTA with tyrosinase.

Given the previous results showing that DTA inhibits tyrosinase activity, we conducted docking studies to understand how DTA binds to and deactivates tyrosinase. We compared the docking scores of kojic acid and arbutin to DTA. The predicted binding affinities (docking scores) between tyrosinase and kojic acid, arbutin, and DTA were −5.7, −5.3 kcal/mol, and −5.7 kcal/mol, respectively (Figure 6a,c,e). To better understand their binding modes with tyrosinase, we employed LigandScout 4.4.8 software for binding mode analysis. Kojic acid demonstrated three hydrogen bond acceptor (HBA) interactions and three hydrogen bond donor (HBD) interactions, stabilizing the ligand within the tyrosinase binding site by both accepting and donating hydrogen bonds with surrounding residues (Figure 6b). Arbutin displayed a complex interaction profile with tyrosinase, including seven HBA interactions, five HBD interactions, one hydrophobic (H) interaction, and one aromatic (AR) interaction, contributing to various stabilizing effects within the binding site (Figure 6d). DTA may exhibit two HBA interactions and one negatively charged interaction (NI), potentially enhancing its binding affinity through electrostatic attraction with positively charged residues in the protein (Figure 6f).

## 3. Discussion

Although previous research indicated that docosatrienoic acid (DTA) accumulate in human fibroblast phospholipids when cultured in a basal nutrient medium (MCDB 110) with reduced fetal bovine serum levels (0.4%), the role of this lipid in skin aging is not well understood. The findings of this study support the anti-melanogenic properties of DTA, showcasing its potential in modulating melanogenesis. Previous studies have highlighted the role of various fatty acids in modulating melanogenesis, often by targeting tyrosinase activity or transcription factors like MITF. For instance, alpha-linolenic acid and linoleic acid, structurally similar to DTA, have been shown to inhibit melanogenesis through both direct and indirect pathways. Our study further supports these findings, demonstrating that DTA also reduces melanin production, primarily through the inhibition of MITF translocation [21].

Melanin pigmentation plays a critical role in protecting the skin from ultraviolet (UV) radiation, as melanin absorbs and dissipates the energy from UV rays, thereby preventing DNA damage in skin cells [22,23]. However, the role of melanin extends beyond simple photoprotection. Melanin is also involved in modulating immune responses, neuroprotection, and regulating reactive oxygen species (ROS) [5,24,25]. These additional roles highlight the importance of maintaining balanced melanin production, as both excessive and insufficient melanin can have significant physiological consequences.

UV radiation, particularly UVB, is a well-established inducer of melanogenesis. UV exposure triggers a cascade of molecular events within melanocytes, leading to increased melanin production as a protective response. However, the effects of UV radiation are not confined to the skin alone [26]. UV exposure influences various biological systems, including the neuroendocrine and immune systems, through mechanisms collectively known as “photo-neuro-immuno-endocrinology” [8]. This complexity underscores the need for a comprehensive understanding of UV radiation’s impact when considering therapeutic strategies aimed at modulating melanogenesis.

Melanogenesis, the process of melanin production in the skin, is a multifaceted and tightly regulated biological pathway involving a complex network of enzymes and regulatory proteins. Central to this process is the MITF, which acts as a master regulator by controlling the expression of key melanogenic enzymes such as tyrosinase, *TRP-1*, and *TRP-2* [7]. These enzymes are essential for the biosynthesis of melanin, the pigment responsible for skin color and protection against UV radiation. Given MITF’s pivotal role, it represents a strategic target for developing anti-melanogenic therapies aimed at treating hyperpigmentation disorders. Our study has provided insights into the molecular mechanisms by which DTA exerts its effects. We found that DTA effectively inhibits the nuclear translocation of MITF, resulting in a marked decrease in the transcription of melanogenic enzymes. This suppression of MITF activity consequently leads to reduced melanin production, highlighting the potential of DTA as a novel therapeutic agent for skin pigmentation disorders.

However, we believe that DTA does not directly inhibit tyrosinase activity. This conclusion is supported by the observation that DTA exhibited only a very mild inhibitory effect on mushroom tyrosinase at a high concentration of 60 μM. In contrast, significant inhibitory effects on cellular tyrosinase activity and melanin content were observed at much lower concentrations, starting from 1 μM. Therefore, we propose that the inhibitory effects of DTA on cellular tyrosinase and melanogenesis are primarily due to its impact on the mRNA expression levels of tyrosinase, likely resulting from the inactivation of MITF. This distinction may be important as it highlights the indirect mechanism by which DTA modulates melanogenesis. The indirect mechanism of action of DTA might offer several advantages in therapeutic applications. Targeting the transcriptional regulation of melanogenesis could provide a longer-lasting effect as it intervenes earlier in the melanogenic pathway. This may reduce the frequency of application needed for therapeutic effectiveness, improving patient compliance and overall treatment outcomes. Moreover, by modulating MITF activity, DTA may also influence other MITF-regulated pathways involved in cell survival and differentiation, potentially offering additional benefits in skin health and regeneration. Further research is needed to fully understand the broader implications of MITF inhibition by DTA. Investigating the long-term effects of DTA on skin cells, including potential impacts on cell viability and the expression of other MITF target genes, will be essential for developing safe and effective skin care treatments. Additionally, exploring the effects of DTA in various skin types and conditions can help tailor therapies to individual needs, enhancing the efficacy and safety of treatments for hyperpigmentation disorders.

The structural resemblance between DTA and other well-documented anti-melanogenic lipids, such as palmitoleic acid, alpha-linolenic acid, and linoleic acid, was a pivotal component of our research. These lipids are known for their capacity to inhibit melanogenesis, and our findings indicate that DTA shares this functional characteristic. This resemblance suggests that DTA might operate through similar biochemical pathways, potentially offering comparable efficacy in reducing melanin production. Utilizing SwissSimilarity for structural and functional predictions highlights the importance of bioinformatics tools in identifying and validating potential bioactive compounds [17]. These tools enable researchers to predict the functional capabilities of new compounds based on structural similarities to known active molecules. In our study, SwissSimilarity provided critical insights that guided our experimental focus, helping to prioritize DTA as a candidate for further investigation. By leveraging these predictions, we were able to streamline the research process, ensuring a more targeted and efficient exploration of DTA’s anti-melanogenic potential. The application of such bioinformatics tools is not only useful for predicting the functional properties of compounds but also for understanding their potential interactions within biological systems.

Although further studies are necessary, DTA may also hold considerable promise for the cosmetic industry, offering benefits that span beyond its anti-melanogenic properties. Fatty acids and their derivatives are fundamental components in cosmetic formulations due to their multifaceted roles in enhancing product stability, functionality, and aesthetic appeal. They are crucial not only for maintaining the skin’s lipid barrier, thus preventing transepidermal water loss and ensuring optimal hydration—key considerations for anti-aging and sensitive skin products—but also for reinforcing the skin’s protective shield against environmental stressors such as UV radiation and pollutants.

In cosmetic formulations, fatty acid derivatives such as esters play an indispensable role in creating emulsions, particularly in formulations like water-in-oil (W/O) and oil-in-water (O/W). These derivatives significantly enhance the sensory attributes and spreadability of products, thereby improving consumer experience and acceptance. Additionally, fatty acid-derived surfactants are crucial in micellar solubilization processes, which are pivotal for increasing the bioavailability and efficacy of hydrophobic active ingredients in aqueous-based formulations. This capability is particularly advantageous for incorporating potent anti-aging compounds like retinoids into skincare products.

Beyond their emulsifying and solubilizing properties, fatty acids serve as effective moisturizers and skin conditioners, contributing to the overall sensory profile and efficacy of cosmetic formulations [27]. They play a key role in maintaining skin suppleness, smoothness, and elasticity—attributes that are essential for anti-aging and moisturizing products. Moreover, fatty acids possess soothing and anti-inflammatory properties, making them suitable for formulations aimed at addressing sensitive or irritated skin conditions. Further investigation is warranted to explore the potential application of DTA in these cosmetic fields. Its structural similarity to other effective anti-melanogenic and skincare-active lipids suggests that DTA could offer similar or complementary benefits in cosmetic formulations. Research into its specific mechanisms of action, compatibility with other ingredients, and efficacy in diverse product formulations will be essential for fully realizing its potential in skincare and cosmetic applications.

## 4. Materials and Methods

### 4.1. Material

Docosatrienoic acid (DTA): DTA was obtained as part of a fatty acid library purchased from Enzo Life Sciences (Catalog No. BML-L1120-0001) The equipment was sourced from Lausen, Switzerland. This library includes a variety of fatty acids, which were screened for potential anti-melanogenic compounds. Enzo Life Sciences is known for providing high-quality biochemical products, and this library has been extensively used in lipid research studies.

Other Reagents and Materials: The mushroom tyrosinase, NaOH, DCFDA, esterase, Trolox, DHR123, penicillamine, and most other reagents used in this study were purchased from Sigma (St. Louis, MO, USA). Additionally, primers for tyrosinase, microphthalmia-associated transcription factor (MITF), tyrosinase-related protein 1 (*TRP*-1), and tyrosinase-related protein 2 (*TRP*-2) were mostly ordered from Bioneer (Daejeon, Republic of Korea). The anti-MITF antibody used for staining was obtained from Santa Cruz Biotechnology (Santa Cruz, CA, USA). The chemical structure images of lipids used in this study were sourced from PubChem.

### 4.2. Structural Analysis Using SwissSimilarity

We utilized the web-based tool SwissSimilarity to identify compounds structurally similar to DTA and predict its potential biological functions. DTA (docosatrienoic acid, PubChem CID 5312557) showed high similarity scores with several fatty acids, including palmitoleic acid (PubChem CID 445638), cis-vaccenic acid (PubChem CID 5282761), ethanolamine oleate (PubChem CID 16759), oleic acid (PubChem CID 445639), alpha-linolenic acid (PubChem CID 5280934), gondoic acid (PubChem CID 5282768), gamolenic acid (PubChem CID 5280933), dihomo-gamma-linolenic acid (PubChem CID 5280581), arachidonic acid (PubChem CID 444899), alpha-hydroxylinoleic acid (PubChem CID 21158511), and linoleic acid (PubChem CID 5280450). The chemical structure images for these similar lipids were obtained from PubChem. Based on these similarities, we hypothesized that DTA might inhibit melanogenesis. To test this hypothesis, we conducted in vitro and cell-free experiments, applying various concentrations of DTA to melanocyte cultures and related cell lines to assess its impact on melanin synthesis and the expression of melanogenesis-related genes.

### 4.3. Cell Line and Culture Condition

The cells used in this study were B16F10 mouse melanoma cells and HS68 human primary fibroblasts. B16F10 cells were cultured in Dulbecco’s Modified Eagle’s Medium (DMEM) supplemented with 10% fetal bovine serum and 1% penicillin/streptomycin, incubated at 37 °C in a 5% CO_2_ atmosphere. HS68 cells were cultured in DMEM supplemented with 10% fetal bovine serum and 1% penicillin/streptomycin under the same conditions (37 °C in a 5% CO_2_ atmosphere).

### 4.4. Cell Viability Assay

B16F10 cells were cultured in a 96-well plate at a seeding density of 5×10^3^; cells/well for 48 h. Subsequently, they were treated with DTA at concentrations ranging from 1 to 100 μM and cultured for 24 h. All wells were incubated with 5 μL of MTS reagent from the Promega MTS assay kit (CellTiter96® AQueous One Solution, Promega Corporation, Madison, WI, USA) for 1 h, and absorbance was measured at 490 nm using a microplate reader (Molecular Devices, San Jose, CA, USA).

HS68 cells were cultured in a 96-well plate at a seeding density of 8 × 10^3^; cells/well for 48 h. They were treated with DTA at the same concentrations (1 to 100 μM) and under the same conditions as the B16F10 cells.

### 4.5. Melanin Contents

B16F10 cells (5 × 10^3^;) treated with DTA were incubated in 1 N NaOH solution at 60 °C for 1 h to induce lysis. The resulting lysates (100 µL each) were then transferred to a 96-well plate. Absorbance readings to measure melanin content were taken at 409 nm using a spectrophotometer. To quantify protein levels in the same samples, 5 µL of each lysate was used for the BCA assay, with absorbance measured at 560 nm. The melanin absorbance values at 409 nm were then normalized to the protein content determined by the BCA assay to ensure accurate comparisons across samples [28,29,30].

### 4.6. Real-Time PCR

After treating B16F10 cells with DTA, we investigated its effects on melanogenesis by analyzing the expression of *tyrosinase*, *TRP-1*, and *TRP-2* genes. Total RNA (1 µg) was extracted using the RiboEXTM extraction kit (GeneAll, Seoul, Republic of Korea), followed by cDNA synthesis using the Primer Script RT Reagent kit (SMART GENE, Daejeon, Republic of Korea). For real-time PCR analysis, 2 µL of cDNA was added to each well of a PCR plate, mixed with 18 µL of buffer containing specific primers for tyrosinase, *TRP-1*, and *TRP-2*. Subsequently, 50 µL of TOPrealTM SYBR Green qPCR PreMIX (Enzynomics, Daejeon, Republic of Korea) was added to each well. Real-time PCR reactions were conducted using the QuantStudioTM 1 Real-Time PCR System (Applied Biosystems, Foster City, CA, USA). Gene expression levels were normalized using β-actin as a housekeeping gene, and ΔΔCt values were calculated for analysis.

The primer sequences are as follows. Tyrosinase: F: 5′-TTCTGCCTTGGCACAGACTT-3′, R: 5′-CTGCCAGGAGGAGAAGAAGG-3′, TRP-1: F: 5′-GCTGCAGGAGCCTTCTTTCTC-3′, R: 5′-GTCATCAGTGCAGACATCGC-3′, TRP-2: F: 5′-CTCAGAGCTCGGGCTCAGTT-3′, R: 5′-CTGCCAGGAGGAGAAGAAGG-3′.

### 4.7. Immunofluorescence Staining

Immunofluorescence staining was performed according to previously established protocols. B16F10 cells were treated with 1 μM and 5 μM of DTA, and then 1 h later, they were treated with 500 nM α-MSH and stimulated for 6 days. Cells were washed twice with PBS and fixed with 4% paraformaldehyde for 15 min. After fixation, the membrane was washed again with PBS and treated with 0.5% Triton X-100 for 5 min to permeabilize the membrane. Next, cells were incubated with MITF 1 antibody (1:100, sc-515925, Santa Cruz Biotechnology, Santa Cruz, CA, USA) overnight in the dark. Cells were washed with PBS and incubated with FSD™ 488-conjugated secondary antibody (1:500, RSA1145, BioActs, Incheon, Republic of Korea) for 1 h. Cell nuclei were counterstained with DAPI (4′,6-diamidino-2-phenylindole dihydrochloride), cells were observed under a fluorescence microscope, and images were taken.

### 4.8. Mushroom Tyrosinase Activity

To measure tyrosinase activity, 10 µL of DTA at various concentrations was added to each well of a 96-well microplate, followed by mixing with 180 µL of phosphate buffer (pH 6.8) containing 1 mM L-Tyrosine. Then, 10 µL of mushroom tyrosinase (1000 units) was added, and the reaction was allowed to proceed in the dark at 37 °C for 30 min. Absorbance at 492 nm was measured using a microplate reader.

### 4.9. CUPRAC Assay

The CUPRAC solution was prepared by mixing CuCl_2_ (10 mM), neocuproine (75 mM) diluted in MeOH, and distilled water in specific ratios. Each sample was added to a 96-well plate at a volume of 2 µL per well. Then, 198 µL of the prepared solution was added to each well. The plate was incubated in a dry oven at 37 °C for 10 min, protected from oxygen and light. After incubation, absorbance was recorded at 450 nm. Ascorbic acid served as the standard.

### 4.10. ONOO^−^ Assay

Peroxynitrite (ONOO^−^) was measured using dihydrorhodamine 123 (DHR 123). DHR 123 was dissolved in PBS to achieve a final concentration of 5 µM. 3-Morpholinosydnonimine (SIN-1), an ONOO^−^ donor, was added to the DHR 123 solution to a final concentration of 1 mM in a test tube. The mixture was incubated in the dark at 37 °C for 30 min. The fluorescence intensity was measured using a fluorescence spectrophotometer with an excitation wavelength of 500 nm and an emission wavelength of 536 nm. An increase in fluorescence intensity indicated the generation of ONOO^−^.

### 4.11. Protein-Ligand Docking Simulation

Protein-ligand docking simulations were executed using AutoDock Vina 1.1.2. Predictions of binding affinity were based on the 3D structure of the tyrosinase protein from Agaricus bisporus (PDB ID: 2Y9X), accessed through the Protein Data Bank. For comparing binding affinity with tyrosinase, arbutin and kojic acid, recognized for their inhibitory effects on tyrosinase, were the primary ligands under investigation. Utilizing the binding site of tropolone as a reference for each protein structure, the tyrosinase binding sites for arbutin (CID: 440936) and kojic acid (CID: 3840) were delineated, with an additional exploration into the potential binding of DTA (HMDB ID: HMDB0002823) to the same site. Ligand structures for arbutin and kojic acid were obtained from PubChem via database searches, while the structure for DTA was obtained from the Human Metabolome Database (HMDB).

### 4.12. Statistical Analysis

Statistical analyses were performed using GraphPad Prism version 5.0 (GraphPad Software, San Diego, CA, USA). All experiments were conducted in triplicate, and data are presented as mean ± standard deviation (SD). Comparisons between multiple groups were made using one-way analysis of variance (ANOVA), followed by Tukey’s post-hoc test for multiple comparisons. All statistical tests were two-tailed, and significance levels were set at *p* < 0.05, *p* < 0.01, and *p* < 0.001, as indicated in the figure legends.

## 5. Conclusions

In summary, this study demonstrates that while DTA does not directly inhibit tyrosinase activity, it significantly impacts melanogenesis by modulating MITF activity and reducing tyrosinase mRNA expression. This modulation leads to a decrease in melanin production, indicating that DTA operates through an indirect mechanism that targets the transcriptional regulation of melanogenesis. These findings highlight DTA’s potential as a novel and targeted approach for skin depigmentation, offering an effective alternative for managing hyperpigmentation disorders.

Moreover, the ability of DTA to influence melanin production through the regulation of MITF and associated pathways suggests that it could provide more sustained and comprehensive effects compared to direct enzyme inhibitors. This indirect mechanism positions DTA as a promising candidate for both therapeutic and cosmetic applications aimed at improving skin aesthetics. These findings lay a strong foundation for further research to explore the practical implementation of DTA in skincare products, particularly for conditions involving excessive pigmentation.

## Data Availability

Data are contained within the article.
